# Identification of children with chronic kidney disease through school urinary screening using urinary protein/creatinine ratio measurement: an observational study

**DOI:** 10.1007/s10157-020-01852-5

**Published:** 2020-01-31

**Authors:** Nobuyuki Kajiwara, Kazuyuki Hayashi, Makoto Fujiwara, Hirofumi Nakayama, Yoshikazu Ozaki

**Affiliations:** 1 Department of Nephrology, Ikeda City Hospital, Ikeda, Osaka Japan; 2 Department of Pediatrics, Osaka University Graduate School of Medicine, Suita, Osaka Japan; 3 The 1st Department of Oral and Maxillofacial Surgery, Osaka University Graduate School of Dentistry, Suita, Osaka Japan; 4 Osaka Regional Center for Japan Environment and Children’s Study, Suita, Osaka Japan; 5 Department of Pediatrics, Ikeda City Hospital, Ikeda, Osaka Japan

**Keywords:** Chronic kidney disease (CKD), Chronic glomerulonephritis, Renal biopsy, Incident rate, School urinary screening, Urinary protein/creatinine ratio

## Abstract

**Background:**

School urinary screening has been performed in Japan.

**Methods:**

Ikeda City and Toyono Town introduced, in 2012 and 2013, urinary protein/creatinine (Cr) ratio measurement into the urine-screening protocols designed for students aged between 4 and 15 years. For each student whose urinary protein/Cr ratio was ≥ 0.15 g/gCr (positive case), an appointment was made with a specialist at Ikeda City Hospital. The results of these screening urinalyses conducted through 2018 are summarized.

**Results:**

14,606 junior high and elementary school students aged between 6 and 15 years were included. On average, they underwent 4.16 screening tests. 77 positive cases were detected, and seven students were diagnosed with high-risk chronic kidney disease (CKD). Of these, four underwent renal biopsy, and two, one, and one were diagnosed with IgA nephropathy, MPGN, and FSGS, respectively. In three students, detection of CKD would have been difficult without urinary screening. Incident rates of high-risk CKD and IgA nephropathy are estimated as 11.5 and 3.3 cases/100,000 students/year. 78.0% of positive cases without high-risk CKD showed no urinary abnormality after one year. 2301 kindergarten students aged between 4 and 6 years received an average of 1.74 screening urinalyses; none was positive or high-risk CKD. The estimated cost of detecting one high-risk CKD student whose detection would have been difficult without this screening was 3,156,711 Japanese yen.

**Conclusion:**

School urinary screening using the urinary protein/Cr ratio can efficiently refer to a specialist. It detects a few children with high-risk CKD early with spending high cost.

**Electronic supplementary material:**

The online version of this article (10.1007/s10157-020-01852-5) contains supplementary material, which is available to authorized users.

## Introduction

In Japan, there has been a consistent decrease in the number of patients who start dialysis therapy due to the progression of chronic glomerulonephritis to end-stage renal disease (ESRD). However, the number of these patients was reportedly as high as 6186 in 2016 [1], which still represents a significant burden to the Japanese healthcare system. School urinary screening has been performed regularly in Japan, Republic of Korea, and Taiwan. Many publications from these countries describe the use of this screening program for successfully identifying children with chronic kidney disease (CKD) [2–5]. In contrast, the American Academy of Pediatrics previously issued a recommendation to discontinue routine dipstick urinalysis for CKD, since this screening procedure is not cost-effective for primary care providers [6].

In previous publications reporting the detection of CKD through school urinary screening, the authors did not clearly describe the post-screening criteria they used for specialist referral of students with abnormal test results. This posed a problem in determining the incidence of CKD in schoolchildren.

In 2006, the Osaka Prefecture public school system introduced the use of urinary protein/creatinine (Cr) ratio measurement into school urinary screening [7]. They also implemented a policy requiring that each student whose urinary protein/Cr ratio was equal to or above a certain value should undergo specialist examination [7]. The Osaka Prefecture public school system consists primarily of senior high schools, largely made up of students aged between 15 and 18 years. Both Ikeda City and Toyono Town are located within Osaka Prefecture. The Ikeda City and Toyono Town public school systems incorporated urinary protein/Cr ratio measurement into school urinary screening in 2012 and 2013, respectively [8]. In this study, the outcome of this new screening strategy performed on students aged between 4 and 15 years was investigated. In addition, the incidence of pediatric CKD was estimated, and the cost performance of the new screening procedure was examined.

## Methods

All students attending municipal public schools underwent screening urinalysis for CKD annually in Ikeda City (from 2012 to 2018) and Toyono Town (from 2013 to 2018). These schools consisted of seven junior high schools, 14 elementary schools, and six kindergartens.

First morning urine was collected. The first screening was performed using the dipstick method. Students who were found to be positive (≥ 1 +) for proteinuria or occult blood (≥ 1 +) and students who did not submit first screening urine were selected for the second urinalysis with the same technique. If they were positive (≥ 1 +) for proteinuria, their urinary protein/Cr ratios were then determined. Students with a urinary protein/Cr ratio of ≥ 0.15 g/gCr were advised to see a specialist. (Until 2015, this cut-off value was set to 0.20 g/gCr. The change of the cut-off value was derived from The Committee of Measures for Pediatric CKD in The Japanese Society for Pediatric Nephrology report that the cut-off value of the urinary protein/Cr ratio in urinary screening is 0.15 g/gCr [9]). For each student, an appointment was made with a nephrologist (junior high school students) or pediatric nephrologist (elementary school and kindergarten students) at Ikeda City Hospital. Separately, each student with occult blood was given a letter, recommending that they see the school doctor or a primary care physician. Letters were sent out even if these students were negative for proteinuria or their urinary protein/Cr ratios were < 0.15 g/gCr.

The results of the school urinary screening conducted during the seven-year study period (from 2012 to 2018) are presented.

### Estimation of the annual incident rate of kidney disease

The number of screening tests each student received during the 7-year study period varied from one to seven. To determine the scale of this study, the actual number of students (junior high school, elementary school, and kindergarten students) who underwent school urinary screening was calculated. The calculation method was as follows (see also Supplementary Figs. 1 and 2). Every year, each school and kindergarten reported the total number of students who underwent screening urinalysis. They did not report the number of students screened for each grade. Therefore, the number of students screened for each year of birth was estimated every year by dividing the total number of students who submitted urine samples by the number of grades in each school (3 for junior high schools, 6 for elementary schools, and 2 for kindergartens). (The total number of students who submitted urine samples was calculated by subtracting the number of students who did not submit for either screening from the total number of students eligible for screening.) Starting in 2013, students who were born in certain specific years began undergoing repeated urinary screening tests. Thus, for each of these birth years, there were multiple different estimates for the number of students screened. (As described above, this number was estimated every year). To determine the actual number of students screened for each year of birth, the mean value of these different estimates was calculated. In Supplementary Figs. 1 and 2, the calculated mean values are shown in italicized red text in the rightmost columns. Data provided by the Ikeda City and Toyono Town Boards of Education demonstrate that only a small number of students move into or out of these municipalities. The data also indicate that few students transfer between a municipal public school and a national or private school when they graduate from their elementary schools and enter junior high schools. Therefore, the numbers of these students were not taken into account in this study. Based on the demographic data publicly available from Ikeda City and Toyono Town, one can conclude that approximately 90% of junior high and elementary school students attend municipal public schools. Thus, the participants of our study represent almost all children aged between 6 and 15 years. School urinary screening is performed annually. Therefore, for each student, the number of screening tests received represents the number of years observed. Consequently, the incident rate of kidney disease per 100,000 junior high and elementary school students can be calculated using the following formula

$$\begin{aligned} &{\text{Annual}}\;{\text{incident}}\;{\text{rate per 1}}00,000{\text{ students }}\left[ {{\text{cases}}/{1}00,000{\text{ students}}/{\text{year}}} \right] \\ &\quad= {\text{Patient number }}\left[ {{\text{cases}}} \right] \times {1}00,000/({\text{Actual number of students screened }}\left[ {{\text{cases}}} \right] \hfill \\ &\qquad \times {\text{Mean number of screening tests per student }}\left[ {{\text{years}}/{\text{case}}} \right]) \hfill \\ \end{aligned}$$

The results presented in this report are primarily raw data and have not been subjected to statistical analyses.

### Cost

Additionally, the cost of school urinary screening to Ikeda City and Toyono Town was calculated. The costs associated with the examinations each student received at Ikeda City Hospital were not determined.

## Results

### Patient number

Table 1 shows the results of school urinary screening conducted at junior high and elementary schools. The table also lists the actions taken based on the screening results and the number of students for each action. During the seven-year study period, a total of 61,144 students were to be examined by school urinary screening. The total number of students who submitted urine samples for the first or second screening was 60,816. Thus, the sample submission rate was 99.5%. The total number of students whose urinary protein/Cr ratios were ≥ 0.20 g/gCr (until 2015) or ≥ 0.15 g/gCr (since 2016) was 77. These students (called “Uprot/Ucr-positive cases” hereafter) were advised to see a specialist at Ikeda City Hospital. In these Uprot/Ucr-positive 77 cases, 68 students saw a specialist at Ikeda City Hospital and 60 students were taken blood sample. All 60 students’ eGFR was higher than 60 mL/min/1.73 m^2^.

**Table 1:** Results of urinary screening, actions taken, and the number of students for each action in junior high and elementary schools

1st Urinalysis		2nd Urinalysis			Direction	Number (high-risk CKD)
Protein	OB	Protein	OB	Uprot/Ucr ratio		
− or ±	− or ±				None	59,183
− or ±	+	+	− or ±	≥ 0.15	Ikeda City Hospital	3
− or ±	+	+	+	≥ 0.15	Ikeda City Hospital	3 (2)
+	− or ±	+	− or ±	≥ 0.15	Ikeda City Hospital	56 (3)
+	− or ±	+	+	≥ 0.15	Ikeda City Hospital	0
+	+	+	− or ±	≥ 0.15	Ikeda City Hospital	0
+	+	+	+	≥ 0.15	Ikeda City Hospital	3 (1)
Not submitted		+	− or ±	≥ 0.15	Ikeda City Hospital	8 (1)
Not submitted		+	+	≥ 0.15	Ikeda City Hospital	0
		+		≥ 0.15	Another hospital	4
Total number of students with a Uprot/Ucr ratio ≥ 0.15 (Uprot/Ucr-positive cases)						77 (7)
OB positive on 1st and/or 2nd urinalysis without a Uprot/Ucr ratio ≥ 0.15					School doctor of family practitioner	600
Protein positive on 1st urinalysis and protein negative or a Uprot/Ucr ratio < 0.15 on 2nd urinalysis without OB					Observation	956
Total number of submitted students						60,816 (7)
Not submitted		Not submitted				328
Total number of corresponded students						61,144 (7)

*OB* occult blood, *Uprot/Ucr ratio* urinary protein/creatinine ratio

As for kindergartens, there were 4036 students to be examined during the 7-year study period. The total number of students who submitted urine samples for the first or second screening was 3996, making the sample submission rate 99.0%. There were no Uprot/Ucr-positive cases among these kindergarten students.

**Fig. 1:**
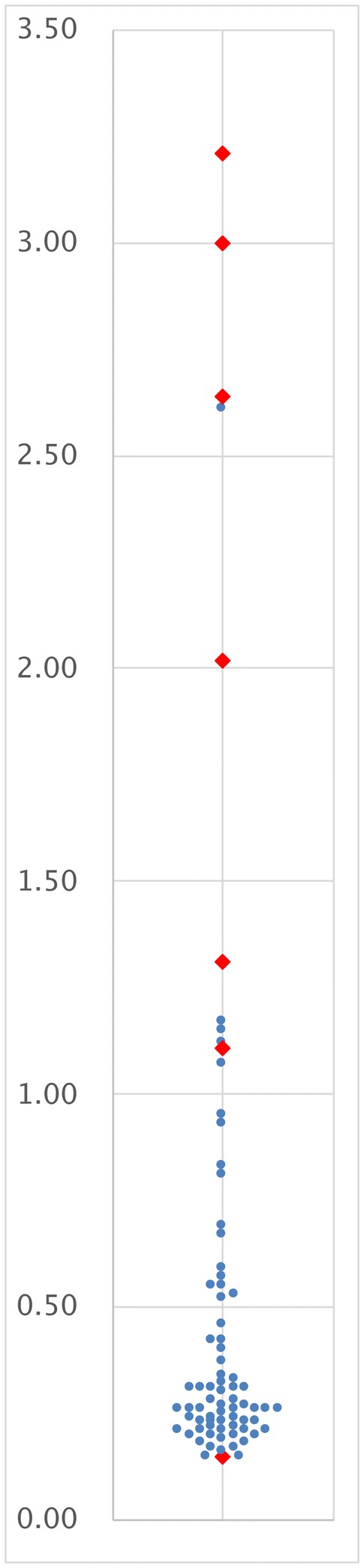
Distribution of the urinary protein/creatinine ratio in 77 students who were advised to see a specialist at Ikeda City Hospital. Red closed diamonds show seven students who were diagnosed as having high risk CKD, and blue closed circles show other children

Table 2 lists the results of detailed examinations performed on Uprot/Ucr-positive cases. All urinary tract infection cases were transient. And, there were no chronic pyelonephritis case. No congenital anomalies of the kidney and urinary tract (called “CAKUT” hereafter) student were newly detected in junior high school, elementary school, and kindergarten. High-risk CKD cases: students undergone renal biopsy and students with nephrotic syndrome or other progressive renal disease were seven. Children whose proteinuria was relatively low or transient, had not undergone renal biopsy, and had not been included in high-risk CKD, posture proteinuria, urinary tract infection, or another criteria were classified as “proteinuria and hematuria syndrome” or “proteinuria syndrome”. These “proteinuria and hematuria syndrome” and “proteinuria syndrome” include CKD for continuation of proteinuria and/or hematuria over three months. The clinical features of these high-risk CKD cases are summarized in Table 3. Four of these students underwent renal biopsy. Of these, a 6-year-old girl with IgA nephropathy had a past history of Henoch–Schönlein purpura, and intermittent macrohematuria appeared. Had school urinary screening not been performed, she would then likely have been diagnosed. Because other students with IgA nephropathy, MPGN, and FSGS showed few objective and subjective symptoms, detection of their CKD would have been difficult without school urinary screening. Two of the high-risk CKD cases were found to have nephrotic syndrome when examined at Ikeda City Hospital. These students were immediately hospitalized, and medication was initiated. They did not undergo renal biopsy. The last student was diagnosed with autosomal dominant polycystic kidney disease. Figure 1 shows the distribution of the urinary protein/Cr ratio in 77 Uprot/Ucr-positive cases. Red closed diamonds show seven high-risk CKD cases, and the blue closed circles show other students. The urinary protein/Cr ratio in six of the seven high-risk CKD cases was over 1.00 g/gCr.

**Table 2:** Diagnostic findings of students with a urinary protein/Cr ratio ≥ 0.15 g/gCr (Uprot/Ucr-positive cases)

Results	Number
High-risk CKD cases	7
Proteinuria and hematuria syndrome (including CKD)	5
Proteinuria syndrome (including CKD)	14
Posture proteinuria	18
Urinary tract infection (transient)	7
Urinary tract infection (transient) and/or acute nephritic syndrome	1
Within normal limit	19
Another hospital	4
Did not come for consultation	2
Total	77

*g/gCr* g/gCreatinine, *CKD* chronic kidney disease

**Table 3:** Detailed characteristics of students diagnosed high-risk CKD cases

Diagnosis	Age (years)	Sex	1st Urinalysis		2nd Urinalysis			eGFRcreat	Treatment
			Protein	OB	Protein	OB	Uprot/Ucr ratio	[mL/min/1.73m^2^]	
IgA nephropathy	6	F	-	3 +	+	3 +	0.15	132.80	PSL + MZ + CyA + warfarin + dipyridamole
IgA nephropathy	7	M	3 +	3 +	3 +	3 +	2.64	99.45	PSL + MZ + warfarin + dipyridamole + ACEI
Membranoproliferative glomerulonephritis	10	F	–	+	3 +	3 +	1.11	84.76	PSL + MMF + CyA + ACEI + CCB
Focal/segmental glomerulosclerosis	12	M	2 +	–	3 +	–	1.31	118.77	PSL + ACEI + ARB + CCB
Nephrotic syndrome (renal biopsy is not performed)	8	M	2 +	–	3 +	–	3.00	134.60	PSL + MZ
Nephrotic syndrome (renal biopsy is not performed)	13	M	3 +	–	3 +	–	3.21		(Another hospital)
Autosomal dominant polycystic kidney disease	12	M	*	*	3 +	–	2.02	119.08	(Close observation)

*CKD* chronic kidney disease, *eGFRcreat* estimated glomerular filtration rate that calculated from creatinine, *OB* occult blood, *Uprot/Ucr ratio* urinary protein/creatinine ratio, *PSL* prednisolone, *MZ* mizoribine, *CyA* cyclosporine, *ACEI* angiotensin-converting-enzyme inhibitor, *MMF* mycophenolate mofetil, *CCB* calcium channel blocker, *ARB* angiotensin receptor blocker

*Not submitted

We did not give a clear direction to students with known renal disease. Though some guardians withheld urinary samples, the number was unclear. In Table 1, “Another hospital” included students with known renal disease.

The following year’s screening data of all Uprot/Ucr-positive cases were collected and analyzed (Table 4). It was not possible to obtain these follow-up data for some of the Uprot/Ucr-positive students. This was partly because they had graduated from their junior high or elementary schools. However, follow-up data were ultimately collected from 48 students, and 41 students had not been confirmed to have high-risk CKD. The analysis of these data demonstrated that 32 (78.0%) of the 41 students showed no abnormality one year after they had a positive Uprot/Ucr.

**Table 4:** Urinalysis data of students whose urinary protein/Cr ratios were ≥ 0.15 g/gCr (Uprot/Ucr-positive cases) in the preceding year’s screening

Results	Number
High-risk CKD cases	7
Uprot/Ucr ratio ≥ 0.15 g/gCr + OB	0
Uprot/Ucr ratio ≥ 0.15 g/gCr	3*
Uprot/Ucr ratio < 0.15 g/gCr + OB	1
Uprot/Ucr ratio < 0.15 g/gCr	5
OB	0
Within normal limit	32
Loss of follow-up (completion)	28
Not submitted	1
Total	77

*Uprot/Ucr ratio* urinary protein/creatinine ratio, *g/gCr* g/gCreatinine, *CKD* chronic kidney disease, *OB* occult blood

*Uprot/Ucr ratios of these students were 0.31, 0.28, 0.15 g/gCr in the follow-up years

Giving a letter recommending that they see the school doctor or a primary care physician to each student with urinary occult blood is a step to alleviate health care teachers’ and guardians’ anxiety. However, no CKD case was reported among the students who had been recommended to see the school doctor or their primary care physician.

### Estimation of the annual incident rate of kidney disease

As described above, the total number of urine samples submitted was 60,816 at junior high and elementary schools. By calculating the sum of the numbers shown in italicized red text in Supplementary Fig. 1 (the rightmost column), the actual number of junior high and elementary school students screened was determined to be 14,606. Based on these, the mean number of screening tests each junior high or elementary school student received during the 7-year study period was estimated as: 60,816/14,606 = 4.16.

Thus, the annual incident rate of high-risk CKD per 100,000 students is 11.5. The annual incident rate of chronic glomerulonephritis (confirmed by renal biopsy) is 6.6/100,000 students. For IgA nephropathy, the annual incident rate is 3.3 cases/100,000 students.

As for kindergarten students, the actual number of students screened was 2301, and the mean number of years observed was 1.74. There was no Uprot/Ucr-positive case or high-risk CKD case. Consequently, the annual incident rate of high-risk CKD per 100,000 kindergarten students should be less than 25.0, as calculated from this: 1 [case] × 100,000/(2301 [cases] × 1.74 [years/case]).

### Costs

The total cost for school urinary screening conducted by Ikeda City and Toyono Town during the seven-year study period was 9,470,132 Japanese yen (¥). Thus, for these local governments, the cost for identifying one high-risk CKD case was: ¥9,470,132/7 [high-risk CKD cases] = ¥1,352,876. The estimated cost of detecting one high-risk CKD case whose detection would have been difficult without school urinary screening was: ¥9,470,132/3 = ¥3,156,711.

## Discussion

In recent years, school urinary screening has been conducted using urinary protein/Cr ratio measurement. However, this is the first report describing the results of this screening procedure that was performed over several years in more than 10,000 children aged between 4 and 15 years.

Previously, we published the results of our preliminary study concerning the school urinary screening performed in 2012 in Ikeda City [8]. In this preliminary study, based on the number of students recommended to have renal biopsy, we estimated that this diagnostic procedure would be indicated in 0.14% of junior high school students annually (140 cases/100,000 students/year). Subsequently, however, proteinuria decreased in all of these students recommended for renal biopsy. As a result, none of them underwent this procedure. There was an overestimation in our preliminary study of the number of students requiring renal biopsy. Utsunomiya et al. [10] reported that, in Yonago City, Japan, the incidence of IgA nephropathy detected by school urinary screening was 4.5 cases/100,000 students/year in students aged between 6 and 15 years. In contrast, Shibano et al. [11] estimated that, in Nishinomiya City, Japan, the incidence of IgA nephropathy detected by school urinary screening was 9.9 cases/100,000 students/year in students aged between 6 and 15 years. One of the indications used by this group for renal biopsy was microscopic hematuria that continued for ≥ 5 years with, or even without, proteinuria. Therefore, their study may have included patients with mild IgA nephropathy, leading to an increase in the estimated incidence of this kidney disease. Ashida et al. [7] reported that the Osaka Prefecture public school system has been using urinary protein/Cr ratio measurement for their urinary screening tests. According to these tests, the incidence of CKD was 5–12 cases/100,000 students/year in their students aged between 15 and 18 years. Based on these publications and the present study, the incidence of pediatric high-risk CKD detected by school urinary screening would be estimated to be approximately 10 cases/100,000 students / year.

In the present study, there was no Uprot/Ucr-positive case or high-risk CKD case in kindergarten students aged between 4 and 6 years. This does not imply that the incidence of high-risk CKD is lower in these students than in older students. Kindergarten is not compulsory in Japan. Furthermore, a relatively large number of children attend private kindergartens in Ikeda City and Toyono Town. Due to these reasons, only a small number of kindergarten students participated in the study. To determine the incidence of CKD in kindergarten students, many more samples would be needed.

In Japan, a 3-year-old health check-up is done for all 3-year-old children, and it includes urinalysis for detecting CAKUT. Ishikura et al. [12] estimated the prevalence of stage 3–5 CKD by CAKUT 2.03 cases/100,000 children from a nationwide, population-based survey of Japanese children aged 3 months–15 years. In this study, the cohort size is about 15,000 children, and there is a possibility that some students with known renal disease withheld urinary samples. Therefore, we think no child with CAKUT is newly detected.

In our current screening program, students with a urinary protein/Cr ratio of ≥ 0.15 g/gCr (i.e., abnormal levels of proteinuria) are directly referred to a pediatric nephrologist or nephrologist. This policy makes the examination process relatively more efficient, since it can eliminate the need for referrals from a primary care physician to a specialist. However, the policy cannot be easily implemented in areas where only a small number of nephrologists are available. One of the approaches to address this problem is to raise the cut-off value of the urinary protein/Cr ratio to 1.00 g/gCr for specialist referrals in those areas. In the present study, most of the Uprot/Ucr-positive cases had a urinary protein/Cr ratio under 1.00 g/gCr. Furthermore, among them, there was only one high-risk CKD case. However, this case showed intermittent macrohematuria after screening. Thus, even if school urinary screening had not been done, she might have been diagnosed.

The incidence of high-risk CKD is low in children. As a result, the cost of detecting one CKD case whose detection would have been difficult without school urinary screening was as high as ¥3,156,711 in our school urinary screening system, and this does not even include the expenses associated with the medical examinations CKD-suspected students needed to receive. To change annual school urinary screening to every two or three years is worthy of consideration for a cost decrease. Sekhar et al. [6] demonstrated that primary care providers can detect one case of CKD per 800 screened, thus incurring a cost of US $2779.50 per case diagnosed. Based on this analysis, they concluded that screening urinalysis is a cost-ineffective procedure for these providers. However, the total annual cost for ESRD therapy (including dialysis) for each patient is approximately ¥4,800,000 in Japan [13], $66,000 in the United States [14], and €39,052 in Greece [15]. Given these high costs, school urinary screening may still be worth the expense, if the early detection of CKD in children helps slow its subsequent progression to ESRD, even if we only detect one child every several years. It remains to be determined whether the detection of CKD in childhood with a screening dipstick urinalysis will lead to effective interventions and alter the future of a child who is destined to ultimately develop ESRD [6]. As compared with the dipstick-only urinalysis, the concomitant use of dipstick and urinary protein/Cr ratio measurement can reduce the cost of school urinary screening without compromising its efficiency [7]. As described above, school urinary screening has been routinely conducted in Japan, Republic of Korea, and Taiwan. Both the incidence and prevalence of ESRD in children are low in Japan and Taiwan [16]. Thus, medical professionals and policymakers involved in children’s health programs should further discuss the use of urinary protein/Cr ratio measurement in school urinary screening procedures and the issue of costs. Furthermore, we recommend that students with urinary occult blood who are negative for proteinuria or their urinary protein/Cr ratios are < 0.15 g/gCr generally have no need to see a doctor.

The main limitation of our research is that only a few students were found to have CKD. However, unlike previous reports, this study allowed us to clearly show the detailed results of the screening tests that led to the identification of students with CKD. In addition, it was possible to carefully examine the urinalysis data of students who had been found to have a positive urinary protein/Cr ratio in the preceding year’s screening. We hope that more school systems will incorporate the use of urinary protein/Cr ratio measurement into their protocols for routine urinalysis. This will enable us to collect more data on the effectiveness of this measurement technique.

In the present study, we demonstrated that urinary screening protocol introduced urinary protein/Cr ratio effectively referred the children with high-risk CKD to the specialists. However, the urinary screening program spent considerable cost to detect few children with high-risk CKD. Although we should keep the program for preventing the progression of the CKD to ESKD in children, we could improve the cost-effectiveness of the program.

## Electronic supplementary material

Below is the link to the electronic supplementary material.

Calculation of the number of junior high and elementary school students screened. The rectangles denote changes in the numbers of junior high (dark blue) and elementary (light blue) school students who submitted urine samples between 2012 and 2018. The vertical and horizontal axes represent the year of birth and year of examination, respectively. The number of students screened was estimated for each year of birth. These numbers are shown in italicized red text in the rightmost column (PDF 2735 kb)

Calculation of the number of kindergarten students screened. The pink rectangles denote changes in the number of kindergarten students who submitted urine samples between 2012 and 2018. The vertical and horizontal axes represent the year of birth and year of examination, respectively. The number of students screened was estimated for each year of birth. These numbers are shown in italicized red text in the rightmost column (PDF 577 kb)
