# Supplementary material for: Identification of children with chronic kidney disease through school urinary screening using urinary protein/creatinine ratio measurement: an observational study
Source: Clin Exp Nephrol. 2020 Jan 31;24(5):450–7. doi: 10.1007/s10157-020-01852-5 (PMC7174251; doi:10.1007/s10157-020-01852-5)
Supplement: Supplementary file 1 — Calculation of the number of junior high and elementary school students screened. The rectangles denote changes in the numbers of junior high (dark blue) and elementary (light blue) school students who submitted urine samples between 2012 and 2018. The vertical and horizontal axes represent the year of birth and year of examination, respectively. The number of students screened was estimated for each year of birth. These numbers are shown in italicized red text in the rightmost column (PDF 2735 kb) [file 10157_2020_1852_MOESM1_ESM.pdf]

Year of birth \ Examination year	2012	2013	2014	2015	2016	2017	2018	Estimated students number in each year of birth
1997	2500							*833.3*
1998		3047						*1015.7*
1999			2993					*1006.6*
2000	5356			2918				*995.2*
2001		6094			2911			*989.0*
2002			5961			2865		*981.4*
2003				5899			2765	*973.2*
2004					5811			*972.9*
2005						5843		*976.0*
2006							5856	*985.1*
2007								*979.0*
2008								*975.3*
2009								*972.8*
2010								*974.9*
2011								*976.0*
