# Supplementary material for: Identification of children with chronic kidney disease through school urinary screening using urinary protein/creatinine ratio measurement: an observational study
Source: Clin Exp Nephrol. 2020 Jan 31;24(5):450–7. doi: 10.1007/s10157-020-01852-5 (PMC7174251; doi:10.1007/s10157-020-01852-5)
Supplement: Supplementary file 2 — Calculation of the number of kindergarten students screened. The pink rectangles denote changes in the number of kindergarten students who submitted urine samples between 2012 and 2018. The vertical and horizontal axes represent the year of birth and year of examination, respectively. The number of students screened was estimated for each year of birth. These numbers are shown in italicized red text in the rightmost column (PDF 577 kb) [file 10157_2020_1852_MOESM2_ESM.pdf]

Year of birth \ Examination year	2012	2013	2014	2015	2016	2017	2018	Estimated students number in each year of birth
2006	388							*194.0*
2007		689						*344.5*
2008			632					*330.1*
2009				629				*315.1*
2010					599			*307.0*
2011						538		*284.1*
2012							523	*265.1*
2013								*261.5*
